# Excessive Exercise Habits in Marathoners as Novel Indicators of Masked Hypertension

**DOI:** 10.1155/2017/1342842

**Published:** 2017-02-15

**Authors:** Young-Joo Kim, Yongbum Park, Duk-Ho Kang, Chul-Hyun Kim

**Affiliations:** ^1^Department of Rehabilitation Medicine, College of Medicine, Sanggye-Paik Hospital, Inje University, Seoul, Republic of Korea; ^2^Department of Sports Medicine, Soonchunhyang University, Chungnam, Republic of Korea

## Abstract

*Background*. Excessive exercise such as marathon running increases the risk of cardiovascular events that may be related to myocardial infarction and sudden death. We aimed to investigate that the exercise characteristics can be used as a novel indicator of masked hypertension.* Methods*. A total of 571 middle-aged recreational male marathoners were assigned to a high blood pressure group (HBPG; *n* = 214) or a normal blood pressure group (NBPG; *n* = 357). A graded exercise test was used to examine the hemodynamic response and cardiac events, and the personal exercise characteristics were recorded.* Results*. Systolic blood pressure and diastolic blood pressure were higher in the HBPG than in the NBPG (*p* < 0.05, all). The marathon history, exercise intensity, and time were longer and higher, whereas the marathon completion duration was shorter in the HBPG than in NBPG (*p* < 0.05, all). HBPG showed a higher frequency of alcohol consumption than NBPG (*p* < 0.05).* Conclusion*. More excessive exercise characteristics than the normative individuals. If the individuals exhibit high blood pressure during rest as well as exercise, the exercise characteristics could be used as a novel indicator for masked hypertension.

## 1. Background

Regular exercise is effective for the prevention and treatment of chronic diseases such as coronary artery disease (CAD), diabetes, obesity, hypertension, cardiac failure, and depression and reportedly extends the average lifespan of active individuals by approximately 7 years, as compared to individuals with a sedentary lifestyle [1–3]. In contrast, excessive exercise that is commonly accumulating workloads of 200 to 300 metabolic equivalent hours (METs × Hours) such as marathon [4] increases the risk of cardiovascular events that may be related to myocardial infarction and sudden death [5, 6]. In athletes aged ≤35 year, the leading cause of sudden death is hypertrophic cardiomyopathy, whereas, among athletes aged >35 years, the leading cause of sudden death is CAD (70%) [7]. The risk of CAD is known to increase during middle-aged, and the primary factors involved include diabetes, hyperlipidemia, smoking, and hypertension [8]. Of these factors, hypertension exhibits the highest prevalence and is observed in nearly 1 of 3 adults (32%) [9]. With regard to the positive effects of exercise on hypertension, systolic and diastolic blood pressure (SBP/DBP) have been found to decrease by 4/4 mmHg in a healthy group and by 11/6 mmHg in a hypertension group [10]. Nevertheless, recent studies have also indicated that excessive exercises such as marathon running actually increase arterial stiffness and can promote the progression of atherosclerosis [11–13]. Moreover, the prevalence of atrial fibrillation and other arrhythmias requiring treatment is known to be higher among individuals who participate in extreme exercise than among typical individuals [14, 15]. In particular, most participants involved in excessive exercise, such as those participating in Ironman competitions, marathons, and 100 km ultramarathons, are middle-aged individuals aged ≥40 years [16–18]. Among these individuals, the increase in arterial stiffness and arrhythmia caused by excessive exercise is considered to be associated with hypertension [11–15]. In the present study, we aimed to assess whether the exercise characteristics in amateur, middle-aged male marathoners with high blood pressure can serve as a novel indicator of masked hypertension. It is hypothesized that the middle-aged male marathoners with high blood pressure will show excessive exercise habits.

## 2. Methods

This is a retrospective case-control study. After receiving approval from the local ethics committee and obtaining patients' written informed consent, data from 610 middle-aged male marathoners (≥40 and <60 years old) who participated in a graded exercise test (GXT) for a comprehensive physical check-up at our institution between January 2011 and January 2016 were obtained via a manual review of the electronic medical records from our institution's electronic health records. All subject underwent a standardized history, physical examination.

**Figure 1:**
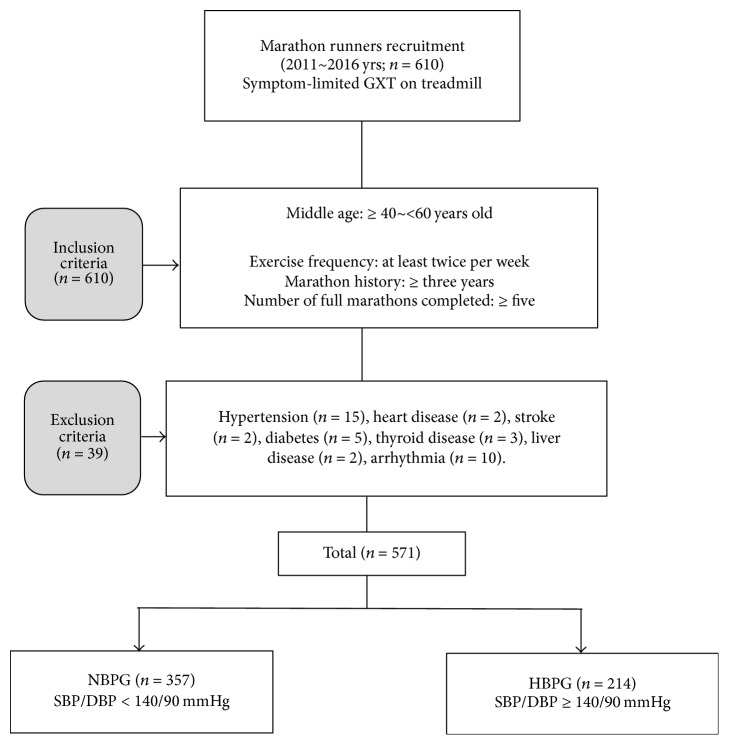
Flow chart of the study procedure. GXT, graded exercise test; NBPG, normal blood pressure group; HBPG, high blood pressure group; SBP, systolic blood pressure; DBP, diastolic blood pressure.

Inclusion criteria included the following: (1) trained at least twice per week for at least 3 consecutive years; (2) completed at least 5 full marathons (42.195 km). Exclusion criteria included the following: (1) taking medication for hypertension (*n* = 15); (2) heart disease (*n* = 2); (3) stroke (*n* = 2); (4) diabetes (*n* = 5); (5) thyroid disease (*n* = 3); (6) liver disease (*n* = 2); (7) arrhythmia (*n* = 10). Among 610 subjects, only 571 subjects met the inclusion and exclusion criteria (Figure 1).

The resting blood pressure (RBP) of all the subjects was recorded at 5-minute intervals during a 10-minute rest period prior to GXT, and the lowest value was used for analysis. The 571 subjects were then assigned to a normal blood pressure group (NBPG; *n* = 214), with a resting SBP/DBP of <140/90 mmHg, or a high blood pressure group (HBPG; *n* = 357), with a resting SBP/DBP of ≥140/90 mmHg. Both groups showed similar values for age, height, weight, body mass index (BMI), and smoking status, whereas alcohol consumption was significantly higher in the HBPG than in the NBPG (*p* < 0.01) (Table 1).

GXT was performed on a treadmill (Medtrack ST 55, Quinton Instrument Co., USA) by using the Bruce protocol. The heart rate and electrocardiogram (ECG) data were recorded using a 12-channel Quinton stress test system (Quinton Q-4500; Quinton Instrument Co., Boston, USA) during the test. The respiratory exchange rates (RER) and maximal oxygen consumption (VO_2max_) measurements were obtained every 15 seconds by using a respiratory gas analyzer (QMC, Quinton Instrument Co., USA). The VO_2max_ was defined as the highest value or the plateau of directly measured VO_2_. RBP measurements were obtained while the subjects sat on a chair in the resting state for 5 minutes, and 2 values were measured with a 5-minute interval; the lowest value was used for analysis. The BP during exercise was recorded during the last minute of each 3-min stage and at the moment of maximum effort, with the arm relaxed at the side (without holding onto the side bar of the treadmill), by using an automatic BP monitor designed for exercise testing (Model 412, Quinton Instrument Co., Boston, USA). To ensure the accuracy of the measurements, the BP values were also obtained manually by placing the stethoscope microphone under the arm cuff and recording the Korotkoff sounds via a head-set. The lowest values were used for analyses. The BP and rate of perceived exertion (RPE) were initially measured 2 minutes after each stage, as well as at 1 minute after the RER of 1 was achieved in each stage. The Borg RPE scale, with values from 6 to 20, was used. The heart rate, ECG data, BP values, and VO_2max_ were recorded during rest and all stages. The recovery period included light walking for 5 minutes at 1.2 mph, according to the Bruce protocol. During this time, the ECG and BP data were assessed at 1-minute intervals, after which all the tests were completed. Pulse pressure (PP) was recorded as the difference between the SBP and DBP. With regard to ST segment depression, upslope depression was defined as a depression of >2 mm, horizontal depression was defined as a depression of >1.4 m, and downslope depression was defined as a depression of >1 mm. When ST depression was observed in at least 2 leads, it was considered to indicate significant myocardial ischemia [19]. The test was discontinued if any subjective symptoms were noted, including chest pain or dizziness, or if any serious cardiac events or abnormal BP responses were observed, based on the guidelines of the American College of Cardiology/American Heart Association [19].

Information on the participating marathoners, such as marathon history (month), marathon completed (number of times), marathon completion durations (min), exercise intensity (Borg's scale), exercise duration (min/day), and exercise frequency (times/week), was obtained through a questionnaire. The subjects were asked to provide the most recent record for the marathon completion duration.

### 2.1. Statistical Analysis

For all the data recorded in the present study, the descriptive statistics (mean, standard deviation) were calculated using the Windows SPSS/PC 17.0 statistics program, whereas between-group differences were tested using an independent *t*-test. Prior to testing the between-group differences, Levene's test was performed to confirm the homogeneity of the variance. With regard to smoking status, ST segment depression, and arrhythmia, the between-group differences in the frequency of these conditions were analyzed using a *χ*^2^ test. The significance level for all statistics was *p* < 0.05.

## 3. Results

The hemodynamic response and cardiorespiratory fitness of all the enrolled subjects are shown in Table 2. The 2 groups showed no difference in HRrest. However, the SBPrest was found to be significantly higher in the HBPG (150.0 ± 10.0 mmHg) than in the NBPG (125.0 ± 9.6 mmHg; *p* < 0.001), whereas the DBPrest was also significantly higher in the HBPG (93.6 ± 10.1 mmHg) than in the NBPG (80.0 ± 7.1 mmHg; *p* < 0.001). Similarly, the HRmax showed no significant difference between the 2 groups. However, the SBPmax was significantly higher in the HBPG (227.9 ± 23.6 mmHg) than in the NBPG (205.6 ± 26.1 mmHg; *p* < 0.001), whereas the DBPmax was also significantly higher in the HBPG (77.8 ± 15.1 mmHg) than in the NBPG (71.5 ± 11.5 mmHg; *p* < 0.001). In addition, the VO_2max_ was significantly higher in the HBPG (50.1 ± 6.9) than in the NBPG (48.6 ± 6.7; *p* < 0.05), and the metabolic equivalent (MET) was also significantly higher in the HBPG (794.9 ± 101.3) than in the NBPG (776.7 ± 103.4; *p* < 0.05). The Re-HR at 1 minute and Re-HR at 2 minutes did not exhibit any significant differences between the groups. However, the PPrest was significantly higher in the HBPG (56.1 ± 11.6) than in the NBPG (44.9 ± 8.0; *p* < 0.001), and the PPmax was also significantly higher in the HBPG (150.3 ± 24.7) than in the NBPG (134.1 ± 27.8; *p* < 0.001). Furthermore, no differences in the frequency of ST segment depression and arrhythmia were observed between the groups (Table 2).

The exercise characteristics of the 2 groups are shown in Table 3. The marathon history was significantly longer in the NBPG (96.4 ± 60.9) than in the HBPG (80.7 ± 44.7; *p* < 0.01), but the number of marathons completed did not significantly differ between the groups. The marathon completion duration was significantly shorter in the HBPG (211.2 ± 28.1) than in the NBPG (217.4 ± 29.6; *p* < 0.05), whereas the exercise intensity was significantly higher in the HBPG (13.3 ± 1.6) than in the NBPG (12.9 ± 1.5; *p* < 0.01). The exercise duration was significantly longer in the HBPG (101.3 ± 38.9) than in the NBPG (93.7 ± 37.0; *p* < 0.05), but the exercise frequency did not significantly differ between the groups (Table 3).

## 4. Discussion

In the present study, we aimed to assess the exercise characteristics and cardiovascular factors of middle-aged male marathoners with masked hypertension. Among the 571 middle-aged male marathoners enrolled, 214 subjects were found to have high BP (mean resting SBP/DBP, 150.0 ± 10.0/93.6 ± 10.1 mmHg) and an SBPmax of ≥210 mmHg (mean, 227.9 ± 23.6 mmHg), which indicated the presence of exercise-induced hypertension (EIH) [20]. EIH is an independent risk factor for cardiocerebrovascular disease and is reported to increase the risk of developing hypertension later in life [21–23]. EIH increases the afterload during exercise, as a result of impaired peripheral vasodilation, thus causing an increase in the BP [24]. These mechanisms can lead to increases in BP to values greater than those in healthy individuals, even during daily exercise, and can accordingly lead to resting hypertension [23].

Moreover, the PP was significantly higher in the HBPG than in the NBPG, both at rest and after maximal exercise. As higher PP is an independent risk factor for stroke, such values could also have a negative effect on cardiocerebrovascular disease [25]. Moreover, in the HBPG, the VO_2max_ and MTE were found to be significantly higher than those in the NBPG. The MET value is obtained by dividing the VO_2max_ by 3.5 mL/kg/min. In fact, Dorn et al. reported that an increase of 1MET through exercise in patients with myocardial infarction reduced the mortality rate by 10%, [26] whereas Myers et al. [27] reported that an increase of 1MET corresponded to a 12% increase in survival. The higher VO_2max_ values in the HBPG than in the NBPG may be explained by their exercise characteristics. Although the NBPG showed a significantly longer marathon history than the HBPG, the HBPG exhibited shorter marathon completion durations, higher exercise intensity, and longer exercise durations, as compared to the NBPG. These findings suggest that excessive exercise intensity and duration can increase the BP at rest and during exercise, irrespective of the marathon history.

Appropriate exercise habits improve the vascular endothelial function and may have a positive impact on BP [28, 29], but excessive exercise such as marathon running can increase the cardiovascular risk [11, 30]. In a study by Vlachopoulos et al., marathoners who participated in chronic excessive running showed higher arterial stiffness (which could rapidly progress to atherosclerosis) than typical individuals who did not exercise [13]. An increase in the arterial stiffness was even reported after a short-term training camp for endurance runners [31]. The potential mechanisms through which excessive exercise causes increases in aortic stiffness are as follows. First, chronic pressure on the aortic wall due to extreme exercise would cause mechanical fatigue of the elastic elements of the aortic wall, thus leading to the rapid progression of fibrosis [32]. Second, repetitive episodes of high-intensity strength or endurance exercise may contribute to an increase in BP, potentially via an increase in sympathetic activity [33, 34].

In general, marathoners participate in 90–300 minutes of intense aerobic exercise daily and accumulate 200–300 MET-hours per week, which is 5–10 times higher than the recommended amount in the guidelines to prevent CAD [1]. Moreover, frequent alcohol consumption is known to be a cause of hypertension, and hence, excessive exercise characteristics and frequent alcohol consumption appear to have a combined effect on not only masked hypertension at rest, but also EIH [35].

This study has several limitations. First, the precise diagnosis of hypertension requires 24 h BP measurements, examinations of arterial stiffness, chest radiography, and cardiac ultrasonography. However, such examinations could not be ensured due to the large sample size. Second, psychological tension and white coat hypertension during GXT may have had an effect on the BP measurements; however, this could not be represented in the analysis. Third, although the approximate amount of alcohol consumption in the runners could be determined, it was not possible to measure the precise amount of alcohol consumption; hence, the volume of alcohol consumed could not be compared between the groups.

According to this study, it is implicated that excessive intense exercise and frequent alcohol consumption in middle-aged recreational marathoners can riskily increase blood pressure during exercise and rest and, therefore, it should be important to prescribe properly the intensity of running and to reduce the frequency of alcohol for individual marathoner's health.

Therefore, it is recommended that middle-aged marathoners should perform the annual diagnosis of graded exercise test for their healthcare.

## 5. Conclusion

Individuals with high BP had a shorter exercise history than those with normal BP. Nevertheless, the individuals with high BP still showed more frequent alcohol consumption, higher EIH, higher exercise intensity, longer exercise duration, higher VO_2max_, and shorter marathon completion durations. Thus, frequent alcohol consumption and excessive exercise are associated with high BP, not only at rest, but also during exercise, and may serve as a new indicator of masked hypertension.

## Figures and Tables

**Table 1:** Subject characteristics.

Factor	NBPG	HBPG	Total	*p* value
	(*n* = 357)	(*n* = 214)	(*n* = 571)	
Age (years)	49.1 ± 7.7	48.8 ± 6.6	49.0 ± 7.3	0.676
Height (cm)	169.9 ± 5.3	169.7 ± 5.6	169.8 ± 5.4	0.602
Weight (kg)	67.9 ± 6.8	67.3 ± 7.9	67.7 ± 7.2	0.333
BMI (kg/m^2^)	23.5 ± 1.9	23.3 ± 2.2	23.4 ± 2.0	0.382
Smoking status (%)	10.5% (34)	8% (16)	8.7% (50)	0.397
Alcohol (days/week)	1.3 ± 1.4	1.8 ± 1.9^*∗∗*^	1.5 ± 1.6	0.002

The values are presented as mean ± standard deviation or percentage (number). NBPG, normal blood pressure group; HBPG, high blood pressure group; SBP, systolic blood pressure; DBP, diastolic blood pressure; BMI, body mass index, ^*∗∗*^significantly different from individuals with normal blood pressure at *p* < 0.01.

**Table 2:** Hemodynamic response and cardiorespiratory fitness of the subject.

Factor	NBPG	HBPG	Total	*p* value
	(*n* = 357)	(*n* = 214)	(*n* = 571)	
HR_rest_ (beats/min)	65.2 ± 10.6	64.6 ± 9.7	65.0 ± 10.3	0.502
SBP_rest_ (mmHg)	125.0 ± 9.6	150.0 ± 10.0^*∗∗∗*^	134.2 ± 15.5	0.000
DBP_rest_ (mmHg)	80.0 ± 7.1	93.6 ± 10.1^*∗∗∗*^	85.1 ± 10.6	0.000
HR_max_ (beats/min)	172.9 ± 13.1	172.2 ± 12.7	172.6 ± 12.9	0.550
SBP_max_ (mmHg)	205.6 ± 26.1	227.9 ± 23.6^*∗∗∗*^	214.0 ± 27.4	0.000
DBP_max_ (mmHg)	71.5 ± 11.5	77.8 ± 15.1^*∗∗∗*^	73.8 ± 13.3	0.000
VO_2max_ (mL/kg/min)	48.6 ± 6.7	50.1 ± 6.9^*∗*^	49.1 ± 6.8	0.013
MTE (sec)	776.7 ± 103.4	794.9 ± 101.3^*∗*^	783.5 ± 102.9	0.040
Re-HR 1 min (beats/min)	144.8 ± 16.7	145.8 ± 16.1	45.2 ± 16.4	0.484
Re-HR 2 min (beats/min)	120.0 ± 15.7	120.3 ± 15.5	120.1 ± 15.6	0.815
PP_rest_ (mmHg)	44.9 ± 8.0	56.1 ± 11.6^*∗∗∗*^	49.1 ± 6.8	0.000
PP_max_ (mmHg)	134.1 ± 27.8	150.3 ± 24.7^*∗∗∗*^	140.2 ± 27.8	0.000
ST segment depression (mm)	10 (2.8%)	4 (1.9%)	14 (2.6%)	0.486
Arrhythmia	11 (3.0%)	3 (1.4%)	14 (2.6%)	0.209

Mean ± SD, NBPG; normal blood pressure group, HBPG; high blood pressure group, HR; heart rate, SBP; systolic blood pressure, DBP; diastolic blood pressure, MTE; maximum time to exhaustion during graded exercise test, Re: recovery time, PP; pulse pressure, ^*∗*^significantly different from NBPG at *p* < 0.05, ^*∗∗∗*^significantly different from NBPG at *p* < 0.001.

**Table 3:** Exercise characteristics of the subjects.

Factor	NBPG	HBPG	Total	*p* value
	(*n* = 357)	(*n* = 214)	(*n* = 571)	
Marathon history (month)	96.4 ± 60.9	80.7 ± 44.7^*∗∗*^	90.6 ± 55.8	0.001
Marathons completed (number of times)	44.4 ± 45.3	43.4 ± 37.8	44.0 ± 42.6	0.805
Marathon completion durations (min)	217.4 ± 29.6	211.2 ± 28.1^*∗*^	214.6 ± 29.2	0.031
Exercise intensity (Borg's RPE scale)	12.9 ± 1.5	13.3 ± 1.6^*∗∗*^	13.0 ± 1.6	0.007
Exercise duration (min/day)	93.7 ± 37.0	101.3 ± 38.9^*∗*^	96.6 ± 37.9	0.021
Exercise frequency (times/week)	3.8 ± 1.3	3.8 ± 1.4	3.8 ± 1.3	0.683

The values are presented as mean ± standard deviation or percentage (number). NBPG, normal blood pressure group; HBPG, high blood pressure group; RPE, rate of perceived exertion; ^*∗*^significantly different from NBPG at *p* < 0.05, ^*∗∗*^significantly different from NBPG at *p* < 0.01.

## Abbreviations

HBPG: High blood pressure group
NBPG: Normal blood pressure group
CAD: Coronary artery disease
SBP: Systolic blood pressure
DBP: Diastolic blood pressure
BP: Blood pressure
GXT: Graded exercise test
RBP: Resting blood pressure
BMI: Body mass index (BMI)
RER: Respiratory exchange rates
VO_2max_: Maximal oxygen consumption
RPE: Perceived exertion
PP: Pulse pressure (PP)
EIH: Exercise-induced hypertension.

## Additional Points

*Availability of Data and Materials*. Raw data supporting the obtained results are available at the corresponding author.
